# Supplementary material for: Coalition-structured governance improves cooperation to provide public goods
Source: Sci Rep. 2020 Jun 8;10:9194. doi: 10.1038/s41598-020-65960-8 (PMC7280206; doi:10.1038/s41598-020-65960-8)
Supplement: Supplementary file 1 — Supplementary Material. [file 41598_2020_65960_MOESM1_ESM.pdf]

Coalition-structured governance improves cooperation to provide public goods – Supplementary Material

Vítor V. Vasconcelos*, Phillip M. Hannam*, Simon A. Levin, and Jorge M. Pacheco

Consider a population of size Z. Let i_C be the number of cooperators (C), i_D the number of defectors (D), i_O the number of outsiders (O) (and the number of member $i_M = i_C + i_D = Z - i_O$). With such definitions, we can set the fraction of cooperators, $x_C = i_C/Z$, of defectors, $x_D = \frac{i_D}{Z}$, of members, $y \equiv \frac{i_C+i_D}{Z}$, and the fraction of cooperators within members, $x \equiv \frac{i_C}{i_C+i_D}$.

Balance of cooperators. The number of cooperators increases when a defector (is chosen and then) changes to cooperator, $x_D\, T^{D\to C}$, or when an outsider becomes a cooperator, $(1-y)T^{O\to C}$. It decreases when a cooperator (is chosen and then) changes to D or O, $x_C(T^{C\to D} + T^{C\to O})$.

Balance of defectors. The number of defectors increases when a cooperator (is chosen and then) changes to defector, $x_C\, T^{C\to D}$, or when an outsider becomes a defector, $(1-y)T^{O\to D}$. It decreases when a defector (is chosen and then) changes to C or O, $x_D(T^{D\to C} + T^{D\to O})$.

Balance of outsiders. The number of outsiders increases when a cooperator (is chosen and then) changes to outsider, $x_C\, T^{C\to O}$, or when a defector becomes an outsider, $x_D T^{D\to O}$. It decreases when an outsider (is chosen and then) changes to C or D, $(1-y)(T^{O\to C} + T^{O\to D})$.

Consider that individuals change strategy when they observe that strategy and then decide based on the computation of the payoff difference between the two strategies, Δf_{AB}. Then, we can write the transition probabilities as:

$T^{D\to C} = yx\, F(\beta\, \Delta f_{DC})$, $T^{C\to D} = y(1-x)\, F(\beta\, \Delta f_{CD})$, $T^{O\to C} = yx\, F(\beta\, \Delta f_{OC})$, $T^{C\to O} = (1-y)\, F(\beta\, \Delta f_{CO})$, $T^{O\to D} = y(1-x)\, F(\beta\, \Delta f_{OD})$, and $T^{D\to O} = (1-y)\, F(\beta\, \Delta f_{DO})$.

For large populations (i.e., ignoring noise terms), we can write the evolution of the number of Cs and Os as

$$\frac{dx_C}{dt} = \frac{d\left(\frac{i_C}{Z}\right)}{dt} = \frac{d(x\,y)}{dt} = (1-x)\,y\,T^{D\to C} + (1-y)T^{O\to C} - x\,y(T^{C\to D} + T^{C\to O}) \text{ and} \quad (1)$$

$$\frac{d\left(\frac{i_O}{Z}\right)}{dt} = \frac{d(1-\,y)}{dt} = x\,y\,T^{C\to O} + (1-x)yT^{D\to O} - (1-y)(T^{O\to C} + T^{O\to D}). \quad (2)$$

Substituting all $T^{A\to B}$ and linearizing $F(x) \approx \text{const} + x/2$, we get the

evolution of the fraction of cooperators:

$$\frac{dx_C}{\beta dt} = x_C\,\frac{1}{2}\big(x_D(\Delta f_{DC} - \Delta f_{CD}) + (1-y)(\Delta f_{OC} - \Delta f_{CO})\big) \quad (3)$$

$$\frac{d(x\,y)}{\beta dt} = \frac{1}{2}yx\big((1-x)\,y\ \ (\Delta f_{DC} - \Delta f_{CD}) + (1-y)\,(\Delta f_{OC} - \Delta f_{CO})\big) \text{ and} \quad (4)$$

the **evolution of the fraction of outsiders:**

$$\frac{d(1-\,y)}{dt} = \frac{1}{2}y\,(1-y)\big(x\,(\Delta f_{CO} - \Delta f_{OC}) + (1-x)\,(\Delta f_{DO} - \Delta f_{OD})\big). \quad (5)$$

Writing for x and y, and incorporating β in the timescale, we get

The **evolution of the fraction of members**:

$$\frac{dy}{dt} = \frac{1}{2} y\,(1-y)\big(x\,(\Delta f_{OC} - \Delta f_{CO}) + (1-x)\,(\Delta f_{OD} - \Delta f_{DO})\big) \text{ and} \tag{6}$$

The **evolution of the fraction of cooperators within the coalitions (members)**:

$$\frac{dx}{dt} = \frac{1}{2} x(1-x)\big(y\,(\Delta f_{DC} - \Delta f_{CD}) + (1-y)(\Delta f_{OC} - \Delta f_{CO} + \Delta f_{DO} - \Delta f_{OD})\big). \tag{7}$$

Informed individuals

If individuals have information about the game they are playing and the configuration of the population, they can compute the payoff they will get if they change the strategy. Let us start by showing that the informed individuals have the dynamics expressed in Figure 1 by computing the gain from switching strategies.

$$\begin{aligned}\Delta f_{DC}^{\text{info}}(i_C, i_D) = f_C(i_C+1, i_D-1) - f_D(i_C, i_D) &= \sum_{k=0}^{N[i_M]-1} P(k; i_M-1, N[i_M]-1, i_C)\big(\Pi_C(kc) - \Pi_D(kc)\big)\\ &= -c + c\sum_{k=0}^{N[i_M]-1} P(k; i_M-1, N[i_M]-1, i_C) R(kc)\left(e\frac{1}{N^{\theta'}} + (1-e)\frac{1}{Z^{\theta}}\right)\\ &\approx -c + c\langle R\rangle\left(e\frac{1}{N^{\theta'}} + (1-e)\frac{1}{Z^{\theta}}\right),\end{aligned} \tag{8}$$

$$\begin{aligned}\Delta f_{CD}^{\text{info}}(i_C, i_D) = f_D(i_C-1, i_D+1) - f_C(i_C, i_D) &= \sum_{k=0}^{N[i_M]-1} P(k; i_M-1, N[i_M]-1, i_C-1)\big(\Pi_D(kc) - \Pi_C(kc)\big)\\ &= c - c\sum_{k=0}^{N[i_M]-1} P(k; i_M-1, N[i_M]-1, i_C-1) R(kc)\left(e\frac{1}{N^{\theta'}} + (1-e)\frac{1}{Z^{\theta}}\right)\\ &\approx c - c\langle R\rangle\left(e\frac{1}{N^{\theta'}} + (1-e)\frac{1}{Z^{\theta}}\right),\end{aligned} \tag{9}$$

$$\begin{aligned}\Delta f_{OC}^{\text{info}} &= f_C(i_C+1, i_D) - f_O(i_C, i_D)\\ &= \sum_{k=0}^{N[i_M+1]-1} P(k; i_M, N[i_M+1]-1, i_C)\Pi_C(kc) - \sum_{k=0}^{N[i_M]} P(k; i_M, N[i_M], i_C)\Pi_O(kc)\\ &= \sum_{k=0}^{N[i_M+1]-1} P(k; i_M, N[i_M+1]-1, i_C) B(kc) e\frac{1}{N[i_M+1]^{\theta'}}\\ &+ c\sum_{k=0}^{N[i_M+1]-1} P(k; i_M, N[i_M+1]-1, i_C) R(kc) e\frac{1}{N[i_M+1]^{\theta'}}\\ &+ \left(\sum_{k=0}^{N[i_M+1]-1} P(k; i_M, N[i_M+1]-1, i_C) B(kc+c) - \sum_{k=0}^{N[i_M]} P(k; i_M, N[i_M], i_C) B(kc)\right)(1-e)\frac{1}{Z^{\theta}}\\ &- c - c_c \approx \langle B\rangle e\frac{1}{N[i_M+1]^{\theta'}} + c\langle R\rangle\left(\frac{1}{N[i_M+1]^{\theta'}} + (1-e)\frac{1}{Z^{\theta}}\right) - c - c_c,\end{aligned} \tag{10}$$

$$
\begin{aligned}
\Delta f_{CO}^{\text{info}} = f_O(i_C - 1, i_D) - f_C(i_C, i_D) \\
&= \sum_{k=0}^{N[i_M-1]} P(k; i_M - 1, N[i_M - 1], i_C - 1)\Pi_O(kc) \\
&- \sum_{k=0}^{N[i_M]-1} P(k; i_M - 1, N[i_M] - 1, i_C - 1)\Pi_C(kc) \\
&= -\left(\sum_{k=0}^{N[i_M]-1} P(k; i_M - 1, N[i_M] - 1, i_C - 1)B(kc + c)\right. \\
&\left. - \sum_{k=0}^{N[i_M-1]} P(k; i_M - 1, N[i_M - 1], i_C - 1)B(kc)\right)(1 - e)\frac{1}{Z^\theta} \\
&- c\sum_{k=0}^{N[i_M]-1} P(k; i_M - 1, N[i_M] - 1, i_C - 1)R(kc)\, e\frac{1}{N[i_M]^{\theta'}} \\
&- \sum_{k=0}^{N[i_M]-1} P(k; i_M - 1, N[i_M] - 1, i_C - 1)B(kc)e\frac{1}{N[i_M]^{\theta'}} + c + c_c \\
&\approx -c\langle R\rangle\left(e\frac{1}{N[i_M]^{\theta'}} + (1 - e)\frac{1}{Z^\theta}\right) - \langle B\rangle e\frac{1}{N[i_M]^{\theta'}} + c + c_c,
\end{aligned}
\tag{11}
$$

$$
\begin{aligned}
\Delta f_{DO}^{\text{info}} = f_O(i_C, i_D - 1) - f_D(i_C, i_D) \\
&= \sum_{k=0}^{N[i_M-1]} P(k; i_M - 1, N[i_M - 1], i_C)\Pi_O(kc) - \sum_{k=0}^{N[i_M]-1} P(k; i_M - 1, N[i_M] - 1, i_C)\Pi_D(kc) \\
&= \sum_{k=0}^{N[i_M-1]} P(k; i_M - 1, N[i_M - 1], i_C)B(kc)(1 - e)\frac{1}{Z^\theta} \\
&- \sum_{k=0}^{N[i_M]-1} P(k; i_M - 1, N[i_M] - 1, i_C)B(kc)\left(e\frac{1}{N^{\theta'}} + (1 - e)\frac{1}{Z^\theta}\right) + c_c \\
&\approx -\langle B(kc)\rangle e\frac{1}{N[i_M]^{\theta'}} + c_c, \text{ and}
\end{aligned}
\tag{12}
$$

$$
\begin{aligned}
\Delta f_{OD}^{\text{info}} = f_D(i_C, i_D + 1) - f_O(i_C, i_D) \\
&= \sum_{k=0}^{N[i_M+1]-1} P(k; i_M, N[i_M + 1] - 1, i_C)\Pi_D(kc) - \sum_{k=0}^{N[i_M]} P(k; i_M, N[i_M], i_C)\Pi_O(kc) \\
&= \sum_{k=0}^{N[i_M+1]-1} P(k; i_M, N[i_M + 1] - 1, i_C)B(kc)\left(e\frac{1}{N^{\theta'}} + (1 - e)\frac{1}{Z^\theta}\right) - c_c \\
&- \sum_{k=0}^{N[i_M]} P(k; i_M, N[i_M], i_C)B(kc)(1 - e)\frac{1}{Z^\theta} \approx \langle B(kc)\rangle e\frac{1}{N[i_M + 1]^{\theta'}} - c_c.
\end{aligned}
\tag{13}
$$

What will actually govern the dynamics is the balance between each of the calculus. The term that defines the changes between cooperators and defectors is

$$\Delta f_{DC}^{\text{info}}(i_C, i_D) - \Delta f_{CD}^{\text{info}}(i_C, i_D) \approx 2c\langle R[C'](\varepsilon_1 + \varepsilon_2) - 1\rangle. \tag{14}$$

So, individuals will change strategy depending on how the average marginal return on cooperation compares with the cost of cooperation.

The term that defines the changes from outsiders to cooperators is

$$\Delta f_{OC}^{\text{info}} - \Delta f_{CO}^{\text{info}} \approx 2c(\langle b\rangle \varepsilon_1 + c\langle R\rangle(\varepsilon_1 + \varepsilon_2) - 1 - \kappa). \tag{15}$$

So, outsiders will join and contribute based on how the excludable benefits of the coalition and the marginal returns compare to the cost of membership and cooperation.

The term that defines the changes between defectors and outsiders is

$$\Delta f_{OD}^{\text{info}} - \Delta f_{DO}^{\text{info}} = f_D(i_C, i_D + 1) - f_O(i_C, i_D) - f_O(i_C, i_D - 1) + f_D(i_C, i_D) \approx 2c(\langle b\rangle \varepsilon_1 - \kappa). \tag{16}$$

So, defectors join when the total excludable benefit is bigger than the cost of the membership.

Overall, we can write what governs cooperation and membership as

$$\frac{dx}{dt} \approx x(1-x)c(\langle R(\varepsilon_1 + \varepsilon_2)\rangle - 1) \text{ and} \tag{17}$$

$$\frac{dy}{dt} \approx y\,(1-y)c\big(\langle b\rangle \varepsilon_1 - \kappa + x\,(c\langle R\rangle(\varepsilon_1 + \varepsilon_2) - 1)\big). \tag{18}$$

Uninformed individuals

Individuals in the population interact and derive average payoffs that depend on the configuration of the population, namely, $f_C(i_C, i_D)$, $f_D(i_C, i_D)$, and $f_O(i_C, i_D)$.

If individuals do not know what game is being played, they can compare their average payoff, in which case, $\Delta f_{AB}^{\text{no info}}(i_C, i_D) = f_B(i_C, i_D) - f_A(i_C, i_D)$. In that case, we can compute the difference in calculus of informed and uninformed players.

The difference when changing from D to C is

$$
\begin{aligned}
\Delta f_{DC}^{\text{no info}}(i_C,i_D) - \Delta f_{DC}^{\text{info}}(i_C,i_D) &= f_C(i_C,i_D) - f_C(i_C+1,i_D-1) \\
&= -\sum_{k=0}^{N[i_M]-1} \big(P(k;i_M-1,N[i_M]-1,i_C) - P(k;i_M-1,N[i_M]-1,i_C-1)\big)\Pi_C(kc) \\
&= -\sum_{k=0}^{N[i_M]-1} \big(P(k;i_M-1,N[i_M]-1,i_C) - P(k;i_M-1,N[i_M]-1,i_C-1)\big)B(kc+c)\Big(e\frac{1}{N^{\theta'}} \\
&\quad + (1-e)\frac{1}{Z^{\theta}}\Big) \\
&= -c\sum_{k=0}^{N[i_M]-1} \big(P(k;i_M-1,N[i_M]-1,i_C) - P(k;i_M-1,N[i_M]-1,i_C-1)\big)R(kc)\Big(e\frac{1}{N^{\theta'}} \\
&\quad + (1-e)\frac{1}{Z^{\theta}}\Big) \\
&\quad - \sum_{k=0}^{N[i_M]-1} \big(P(k;i_M-1,N[i_M]-1,i_C) - P(k;i_M-1,N[i_M]-1,i_C-1)\big)B(kc)\Big(e\frac{1}{N^{\theta'}} \\
&\quad + (1-e)\frac{1}{Z^{\theta}}\Big).
\end{aligned} \tag{19}
$$

The difference when changing from C to D is

$$
\begin{aligned}
\Delta f_{CD}^{\text{no info}}(i_C,i_D) - \Delta f_{CD}^{\text{info}}(i_C,i_D) &= f_D(i_C,i_D) - f_D(i_C-1,i_D+1) \\
&= \sum_{k=0}^{N[i_M]-1} \big(P(k;i_M-1,N[i_M]-1,i_C) - P(k;i_M-1,N[i_M]-1,i_C-1)\big)\Pi_D(kc) \\
&= \sum_{k=0}^{N[i_M]-1} \big(P(k;i_M-1,N[i_M]-1,i_C) - P(k;i_M-1,N[i_M]-1,i_C-1)\big)B(kc)\Big(e\frac{1}{N^{\theta'}} \\
&\quad + (1-e)\frac{1}{Z^{\theta}}\Big).
\end{aligned} \tag{20}
$$

The difference when changing from O to C is

$$
\begin{aligned}
\Delta f_{OC}^{\text{no info}} - \Delta f_{OC}^{\text{info}} &= f_C(i_C,i_D) - f_C(i_C+1,i_D) \\
&= \sum_{k=0}^{N[i_M]-1} P(k;i_M-1,N[i_M]-1,i_C-1)\Pi_C(kc) - \sum_{k=0}^{N[i_M+1]-1} P(k;i_M,N[i_M+1]-1,i_C)\Pi_C(kc) \\
&= c\Bigg(\sum_{k=0}^{N[i_M]-1} P(k;i_M-1,N[i_M]-1,i_C-1)R(kc)\Big(e\frac{1}{N[i_M]^{\theta'}} + (1-e)\frac{1}{Z^{\theta}}\Big) \\
&\quad - \sum_{k=0}^{N[i_M+1]-1} P(k;i_M,N[i_M+1]-1,i_C)R(kc)\Big(e\frac{1}{N[i_M+1]^{\theta'}} + (1-e)\frac{1}{Z^{\theta}}\Big)\Bigg) \\
&\quad + \Bigg(\sum_{k=0}^{N[i_M]-1} P(k;i_M-1,N[i_M]-1,i_C-1)B(kc)\Big(e\frac{1}{N[i_M]^{\theta'}} + (1-e)\frac{1}{Z^{\theta}}\Big) \\
&\quad - \sum_{k=0}^{N[i_M+1]-1} P(k;i_M,N[i_M+1]-1,i_C)B(kc)\Big(e\frac{1}{N[i_M+1]^{\theta'}} + (1-e)\frac{1}{Z^{\theta}}\Big)\Bigg).
\end{aligned} \tag{21}
$$

The difference when changing from C to O is

$$\begin{aligned}
\Delta f_{CO}^{\text{no info}} - \Delta f_{CO}^{\text{info}} &= f_O(i_C, i_D) - f_O(i_C - 1, i_D) \\
&= \sum_{k=0}^{N[i_M]} P(k; i_M, N[i_M], i_C)\Pi_O(kc) - \sum_{k=0}^{N[i_M-1]} P(k; i_M - 1, N[i_M - 1], i_C - 1)\Pi_O(kc) \\
&= \sum_{k=0}^{N[i_M]} P(k; i_M, N[i_M], i_C)B(kc)\,(1-e)\frac{1}{Z^\theta} \\
&- \sum_{k=0}^{N[i_M-1]} P(k; i_M - 1, N[i_M - 1], i_C - 1)B(kc)\,(1-e)\frac{1}{Z^\theta}.
\end{aligned} \tag{22}$$

The difference when changing from D to O is

$$\begin{aligned}
\Delta f_{DO}^{\text{no info}} - \Delta f_{DO}^{\text{info}} &= f_O(i_C, i_D) - f_O(i_C, i_D - 1) \\
&= \sum_{k=0}^{N[i_M]} P(k; i_M, N[i_M], i_C)\Pi_O(kc) - \sum_{k=0}^{N[i_M-1]} P(k; i_M - 1, N[i_M - 1], i_C)\Pi_O(kc) \\
&= \sum_{k=0}^{N[i_M]} P(k; i_M, N[i_M], i_C)B(kc)(1-e)\frac{1}{Z^\theta} \\
&- \sum_{k=0}^{N[i_M-1]} P(k; i_M - 1, N[i_M - 1], i_C)B(kc)(1-e)\frac{1}{Z^\theta}.
\end{aligned} \tag{23}$$

And the difference when changing from O to D is

$$\begin{aligned}
\Delta f_{OD}^{\text{no info}} - \Delta f_{OD}^{\text{info}} &= f_D(i_C, i_D) - f_D(i_C, i_D + 1) \\
&= \sum_{k=0}^{N[i_M]-1} P(k; i_M - 1, N[i_M] - 1, i_C)\Pi_D(kc) - \sum_{k=0}^{N[i_M+1]-1} P(k; i_M, N[i_M + 1] - 1, i_C)\Pi_D(kc) \\
&= \sum_{k=0}^{N[i_M]-1} P(k; i_M - 1, N[i_M] - 1, i_C)B(kc)\left(e\frac{1}{N^{\theta'}} + (1-e)\frac{1}{Z^\theta}\right) \\
&- \sum_{k=0}^{N[i_M+1]-1} P(k; i_M, N[i_M + 1] - 1, i_C)B(kc)\left(e\frac{1}{N^{\theta'}} + (1-e)\frac{1}{Z^\theta}\right).
\end{aligned} \tag{24}$$

Thus, we can compute the terms that govern the balance of cooperators within the coalitions as

$$
\begin{aligned}
&\Delta f_{DC}^{\text{no info}} - \Delta f_{CD}^{\text{no info}} \\
&\qquad = \Delta f_{DC}^{\text{info}} - \Delta f_{CD}^{\text{info}} \\
&\qquad - c \sum_{k=0}^{N[i_M]-1} \big(P(k; i_M - 1, N[i_M] - 1, i_C) - P(k; i_M - 1, N[i_M] - 1, i_C - 1)\big) R(kc) \left(e \frac{1}{N^{\theta'}}\right. \\
&\qquad \left. + (1-e)\frac{1}{Z^{\theta}}\right) \\
&\qquad - 2 \sum_{k=0}^{N[i_M]-1} \big(P(k; i_M - 1, N[i_M] - 1, i_C) - P(k; i_M - 1, N[i_M] - 1, i_C - 1)\big) B(kc) \left(e \frac{1}{N^{\theta'}}\right. \\
&\qquad \left. + (1-e)\frac{1}{Z^{\theta}}\right),
\end{aligned} \tag{25}
$$

which reveals a cost to cooperation when changing from D to C and vice versa, and

$$
\begin{aligned}
&\Delta f_{OC}^{\text{no info}} - \Delta f_{CO}^{\text{no info}} + \Delta f_{DO}^{\text{no info}} - \Delta f_{OD}^{\text{no info}} \\
&\qquad = \Delta f_{OC}^{\text{info}} - \Delta f_{CO}^{\text{info}} + \Delta f_{DO}^{\text{info}} - \Delta f_{OD}^{\text{info}} \\
&\qquad - c \sum_{k=0}^{N[i_M+1]-1} P(k; i_M, N[i_M + 1] - 1, i_C) R(kc) \left(e \frac{1}{N[i_M + 1]^{\theta'}} + (1-e)\frac{1}{Z^{\theta}}\right) \\
&\qquad + c \sum_{k=0}^{N[i_M]-1} P(k; i_M - 1, N[i_M] - 1, i_C - 1) R(kc) \left(e \frac{1}{N[i_M]^{\theta'}} + (1-e)\frac{1}{Z^{\theta}}\right) \\
&\qquad - \sum_{k=0}^{N[i_M]-1} \big(P(k; i_M - 1, N[i_M] - 1, i_C) - P(k; i_M - 1, N[i_M] - 1, i_C - 1)\big) B(kc) \left(e \frac{1}{N[i_M]^{\theta'}}\right. \\
&\qquad \left. + (1-e)\frac{1}{Z^{\theta}}\right) \\
&\qquad - \sum_{k=0}^{N[i_M-1]} \big(P(k; i_M - 1, N[i_M - 1], i_C) - P(k; i_M - 1, N[i_M - 1], i_C - 1)\big) B(kc) (1-e) \frac{1}{Z^{\theta}},
\end{aligned} \tag{26}
$$

which reveals another cost to cooperation when moving in and out of the coalition. These costs of no information are a function of the difference in the distribution of cooperators. Thus, we define the cost of cooperation as K with the following interpretation.

The **evolution of the fraction of cooperators within the members in a population of uninformed individuals** is

$$\frac{dx^{\text{no info}}}{dt} = x(1-x)(f_C - f_D). \tag{27}$$

Thus, we are going to set K as a term that reduces the payoff of cooperators as in

$$\frac{dx^{\text{no info}}}{dt} = x(1-x)\Bigg(\underbrace{f_C - f_D + \mathrm{K}}_{\text{informed players calculus}} - \mathrm{K}\Bigg). \tag{28}$$

Identically for the group membership cost. The **evolution of the fraction of members in a population of uninformed individuals** is

$$\frac{dy^{\text{no info}}}{dt} = y\,(1-y)(x\,f_C + (1-x)\,f_D - f_O) \text{ and} \tag{29}$$

so we write it as a cost to membership as in

$$\frac{dy^{\text{no info}}}{dt} = y\,(1-y)\left(\underbrace{x\,f_C + (1-x)\,f_D - f_O + K_M}_{\text{informed players calculus}} - K_M\right). \tag{30}$$

For completeness, we compute the terms K and K_M by plugging in the expressions above.

We get that

$$\begin{aligned}
2\mathrm{K} = {} & y\left(\Delta f_{DC}^{\text{info}} - \Delta f_{CD}^{\text{info}}\right) + (1-y)\left(\Delta f_{OC}^{\text{info}} - \Delta f_{CO}^{\text{info}} + \Delta f_{DO}^{\text{info}} - \Delta f_{OD}^{\text{info}}\right) \\
& - \left(\mathrm{y}\left(\Delta f_{DC}^{\text{no info}} - \Delta f_{CD}^{\text{no info}}\right) + (1-y)\left(\Delta f_{OC}^{\text{no info}} - \Delta f_{CO}^{\text{no info}} + \Delta f_{DO}^{\text{no info}} - \Delta f_{OD}^{\text{no info}}\right)\right) \\
& = y\left(\left(\sum_{k=0}^{N[i_M]-1}\left(P(k; i_M - 1, N[i_M]-1, i_C)B(kc+c)\right.\right.\right. \\
& \left.\left. - P(k; i_M - 1, N[i_M]-1, i_C - 1)B(kc)\right)\right)\left(e\frac{1}{N[i_M]^{\theta'}} + (1-e)\frac{1}{Z^{\theta}}\right) \\
& - \sum_{k=0}^{N[i_M+1]-1} P(k; i_M, N[i_M+1]-1, i_C)R(kc)c\left(e\frac{1}{N[i_M+1]^{\theta'}} + (1-e)\frac{1}{Z^{\theta}}\right) \\
& \left. - \sum_{k=0}^{N[i_M-1]}\left(P(k; i_M - 1, N[i_M-1], i_C) - P(k; i_M - 1, N[i_M-1], i_C - 1)\right)B(kc)(1-e)\frac{1}{Z^{\theta}}\right) \\
& + \sum_{k=0}^{N[i_M+1]-1} P(k; i_M, N[i_M+1]-1, i_C)R(kc)c\left(e\frac{1}{N[i_M+1]^{\theta'}} + (1-e)\frac{1}{Z^{\theta}}\right) \\
& + \left(\sum_{k=0}^{N[i_M]-1}\left(P(k; i_M - 1, N[i_M]-1, i_C)B(kc)\right.\right. \\
& \left.\left. - P(k; i_M - 1, N[i_M]-1, i_C - 1)B(kc+c)\right)\right)\left(e\frac{1}{N[i_M]^{\theta'}} + (1-e)\frac{1}{Z^{\theta}}\right) \\
& + \sum_{k=0}^{N[i_M-1]}\left(P(k; i_M - 1, N[i_M-1], i_C) - P(k; i_M - 1, N[i_M-1], i_C - 1)\right)B(kc)(1-e)\frac{1}{Z^{\theta}}
\end{aligned} \tag{31}$$

and

$$
\begin{aligned}
2\mathrm{K_M} &= x\left(\Delta f_{OC}^{\text{info}} - \Delta f_{CO}^{\text{info}}\right) + (1-x)\left(\Delta f_{OD}^{\text{info}} - \Delta f_{DO}^{\text{info}}\right) \\
&\quad - \left(x\left(\Delta f_{OC}^{\text{no info}} - \Delta f_{CO}^{\text{no info}}\right) + (1-x)\left(\Delta f_{OD}^{\text{no info}} - \Delta f_{DO}^{\text{no info}}\right)\right) \\
&= x\left(\sum_{k=0}^{N[i_M-1]} \big(P(k; i_M-1, N[i_M-1], i_C) - P(k; i_M-1, N[i_M-1], i_C-1)\big)B(kc)\,(1-e)\frac{1}{Z^\theta}\right. \\
&\quad - \sum_{k=0}^{N[i_M]-1} \big(P(k; i_M-1, N[i_M]-1, i_C-1)B(kc+c) \\
&\quad - P(k; i_M-1, N[i_M]-1, i_C)B(kc)\big)\left(e\frac{1}{N[i_M]^{\theta'}} + (1-e)\frac{1}{Z^\theta}\right) \\
&\quad \left. + \sum_{k=0}^{N[i_M+1]-1} P(k; i_M, N[i_M+1]-1, i_C)cR(kc)\left(e\frac{1}{N[i_M+1]^{\theta'}} + (1-e)\frac{1}{Z^\theta}\right)\right) \\
&\quad + \sum_{k=0}^{N[i_M]} P(k; i_M, N[i_M], i_C)B(kc)\,(1-e)\frac{1}{Z^\theta} \\
&\quad - \sum_{k=0}^{N[i_M-1]} P(k; i_M-1, N[i_M-1], i_C)B(kc)\,(1-e)\frac{1}{Z^\theta} \\
&\quad - \sum_{k=0}^{N[i_M]-1} P(k; i_M-1, N[i_M]-1, i_C)B(kc)\left(e\frac{1}{N[i_M]^{\theta'}} + (1-e)\frac{1}{Z^\theta}\right) \\
&\quad + \sum_{k=0}^{N[i_M+1]-1} P(k; i_M, N[i_M+1]-1, i_C)B(kc)\left(e\frac{1}{N[i_M+1]^{\theta'}} + (1-e)\frac{1}{Z^\theta}\right).
\end{aligned}
\tag{32}
$$

K and K_{M} as a function of the structure

Let us consider the limiting case when there is the least structure, i.e., no overlap: $\alpha = 1$. Then, $N[z] = z$ and $P(k; i, i, i_C) = \delta_{k\, i_C}$. So, we get

$$
\begin{aligned}
2\mathrm{K} &= y\, c\big(R(i_C c - c) + R(i_C c)\big)\left(e\frac{1}{{i_M}^{\theta'}} + (1-e)\frac{1}{Z^\theta}\right) \\
&\quad + c\left(R(i_C c)\left(e\frac{1}{(i_M+1)^{\theta'}} + (1-e)\frac{1}{Z^\theta}\right) + R(i_C c - c)(1-e)\frac{1}{Z^\theta}\right)(1-y)
\end{aligned}
\tag{33}
$$

which, noticing that,

$$
\frac{dx^{\text{no info}}}{dt} = x(1-x)\left(\underbrace{-c + \mathrm{K}}_{\text{informed players calculus}} - \mathrm{K}\right) \tag{34}
$$

shows how individuals lose the ability to capture the marginal returns to cooperation when there is only a single coalition, independently of its size (large or small coalition). Identical for membership, where the is a positive term sense they lose the ability to recognize how there share of the excludable good will be smaller:

$$
2\mathrm{K_M} = xc\left(\big(R(i_C c) + R(i_C c - c)\big)(1-e)\frac{1}{Z^\theta} + R(i_C c)e\frac{1}{(i_M+1)^{\theta'}}\right) - B(i_C c)\left(e\frac{1}{{i_M}^{\theta'}} - e\frac{1}{(i_M+1)^{\theta'}}\right) \text{ and} \tag{35}
$$

$$\frac{dy^{\text{no info}}}{dt} = y\,(1-y)\left(\underbrace{x\,c\left(R(i_C c)\left(e\frac{1}{{i_M}^{\theta'}}+(1-e)\frac{1}{Z^{\theta}}\right)-1\right)+B(i_C c)e\frac{1}{{i_M}^{\theta'}}-c_C+K_M}_{\text{informed players calculus}}-K_M\right). \qquad (36)$$

The opposite extreme case, when there are the most overlapping coalitions, is when the coalition size is restricted the most and the population is very large. So, we take $\alpha \to \infty$ and $Z \to \infty$ and we scale $g_m Z \to n$ and, for simplicity, let θ be a positive fixed number. In that case, $N[x] \to n$ and P becomes a function of the fraction of cooperators withing the coalitions, $x = i_C/i_M$, $P(k; i_M, n, i_C) \to P(k; n, i_C/i_M)$. Then,

$$\mathrm{K} = 0 \text{ and } \mathrm{K_M} = 0, \qquad (37)$$

showing how the overlap removes the costs of lack of information.

Figure S 1 below shows intermediate values of population size and overlapping structures. For comparison, we show restricted areas of the phase space (not the full dynamics) that encompass coalitions of the same size ($N[i_M] = 10$). In the main text we represent an example of the complete dynamics.

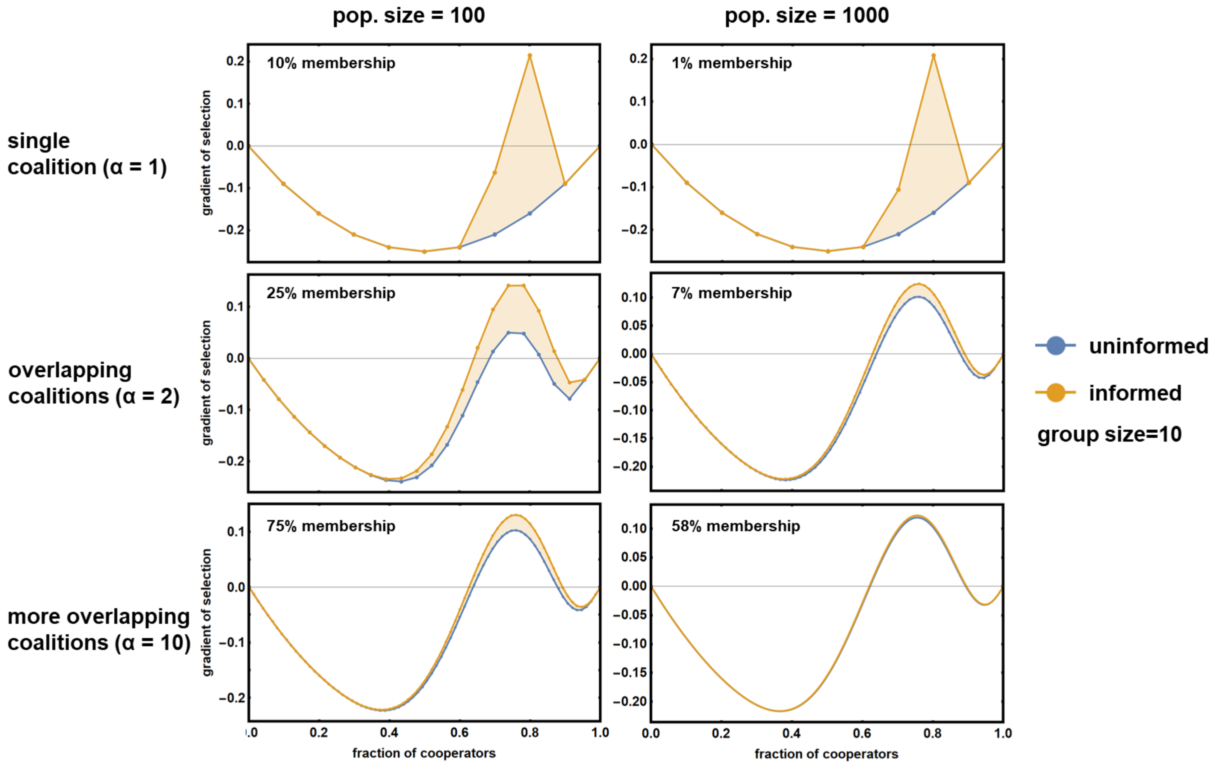

Figure S 1. The panels show the effect of the overlap of coalitions in a slice of the gradient of selection with a fixed group size ($N = 10$) for two different population sizes. It shows that the effect of the structure of interaction exists independently of the group size. This slice does not represent the full dynamics, which are represented in the main text as it takes a fixed membership values, which is a dynamics component. As alpha increases, uninformed populations behave as informed population, being able to access the marginal gains of cooperation. In orange we plot $\dot{x} = x(1-x)(f_C - f_D + K)$ and in blue $\dot{x} = x(1-x)\big((f_C - K) - f_D + K\big) = x(1-x)(f_C - f_D)$. Same parameters as in Figure 2.
